# Impact of alternative approaches to assess outlying and influential observations on health care costs

**DOI:** 10.1186/2193-1801-2-614

**Published:** 2013-11-18

**Authors:** Thomas Weichle, Denise M Hynes, Ramon Durazo-Arvizu, Elizabeth Tarlov, Qiuying Zhang

**Affiliations:** Center of Innovation for Complex Chronic Healthcare, Edward Hines, Jr. VA Hospital, Hines, IL 60141 USA; VA Information Resource Center, Edward Hines, Jr. VA Hospital, Hines, IL 60141 USA; Department of Medicine and Center for Clinical and Translational Sciences, College of Medicine, University of Illinois at Chicago, Chicago, IL 60612 USA; Department of Health Policy Administration, School of Public Health, University of Illinois at Chicago, Chicago, IL 60612 USA; Department of Preventive Medicine & Epidemiology, Stritch School of Medicine, Loyola University Chicago, Maywood, IL 60153 USA

**Keywords:** Health care costs, Outliers, Influential observations, Episode of care, Colon cancer

## Abstract

The distributions of medical costs are often skewed to the right because small numbers of patients use large amounts of health care resources. Using data from a study of colon cancer costs, we show, by example, the impact and magnitude of outliers and influential observations on health care costs and compared the effects of statistical costing methods for addressing the disproportionate influence of outliers and influential observations. We used data from a retrospective cohort study of 3,842 elderly veterans with colon cancer who were enrolled in and used health care from, both the Department of Veterans Affairs and Medicare in 1999–2004. After calculating the average colon cancer episode cost and distribution for the full cohort, we used box-plot methods, Winsorization, DFBETAs, and Cook's distance to identify and assess or adjust the outlying and/or influential observations. The number of observations identified as outlying and/or influential ranged from 13 when the predicted DFBETA measurement was greater than 0.15 and the observation was a qualified box-plot outlier to 384 cases using the Winsorization method at the 5th and 95th percentiles. Average costs of colon cancer episodes using these methods were similar. The method of choice from the results of this particular analysis can be conditionally based on whether the purpose is to control only for influential observations or to simultaneously control for outliers and influential observations. Understanding how estimates could change with each approach is important in assessing the impact of a particular method on the results.

## Introduction

Determining the costs of episodes of medical care is an important step in making policy decisions about allocating health care resources. However, as has been well documented in the literature, accurately estimating costs is challenging due to right skewing when small numbers of patients use larger amounts of health care resources than most other patients (Mullahy 1998). In 2009, for example, 22% of total health care expenditures in the United States were allocated to just 1% of the U.S. population, and almost 50% of health care spending was devoted to 5% of the population (Cohen and Yu 2012). In addition, no single estimator is appropriate for all of the processes typically used to generate health care costs data (Basu et al. 2011). The data values for patients at the extreme ends of the value range do not represent the typical experience and can disproportionately influence statistical point estimates. The lack of symmetry, or skewness that is frequently observed in medical cost data, is characterized by these extreme values, known as outliers.

Statistical procedures are useful to identify cases that have deviated from other cases in the sample, resulting in skewness in large datasets. Some of the statistical techniques are nonparametric and avoid assumptions that the data are represented by a particular statistical distribution.

In the medical literature, outliers are often identified by selecting data on patients with the highest costs based on statistical trimming rules (Gregori et al. 2009). Researchers often use cutoff levels ranging from the upper 0.5% to 20% of the cost distribution, for example. Other approaches include selecting outliers based on the geometric mean plus one or more standard deviations or the interquartile method (Cots et al. 2003; Pirson et al. 2006). The arithmetic mean is then calculated based on the data that remain after the outliers have been trimmed. Disadvantages of these approaches are that the analysis results are relevant only to the sample used and the findings cannot be compared to those of other studies.

In addition to identifying outlying cases in a sample, investigators frequently identify observations that are influential. An influential observation is a type of outlying observation whose exclusion results in major changes in the fitted regression function or parameters. Usually, observations exhibiting high leverage (potential to influence regression results) and large residual (in absolute value) are influential. Although all influential observations are outliers, not all outliers are influential observations.

Standard linear regression models are often used to predict average costs for patients because these models are easy to use and their results are easy to interpret. However, these models are based on the assumption that the regression errors have a normal distribution and linear relationships (Paddock et al. 2004; Barber and Thompson 2004). When these assumptions are violated, as in data on costs of episodes of care with values that are markedly different from the rest of the sample, these models are not appropriate.

Generalized linear models (GLMs) can accommodate skewness in large datasets by weighting variances (Blough and Ramsey 2000). Using these models involves specifying an appropriate model for the mean of the outcome variable and the correct mean-variance relationship (variance function) (Mihaylova et al. 2011). Parameters are then estimated after these structural assumptions are taken into consideration. The mean function estimates from GLMs are generally robust, and GLMs are less sensitive than linear regression models to outliers and/or influential observations. However, mis-specifying the variance function in GLMs could result in losses of precision. Also, GLMs can lose efficiency if the data have a large log-scale error variance or the distribution of errors on the log scale is symmetrical but has a heavy tail (Manning and Mullahy 2001; Mihaylova et al. 2011).

Several statistical techniques can be used to identify and address outlying and/or influential cases in highly skewed cost datasets, potentially improving the precision and efficiency of GLMs. Techniques to assess outliers include box-plot analysis (interquartile method), which involves the use of distributional characteristics to identify outliers (Pirson et al. 2006). Winsorization can be used to transform the costs of outlier episodes so that they are equal to a pre-established percentile of the data (Thomas and Ward 2006). For example, if the maximum percentile is set at 95% and the minimum at 5%, Winsorization transforms costs for patients with costs above the 95^th^ percentile to the costs of patients in the 95^th^ percentile and those with costs in the bottom 5% to the costs of patients in the 5^th^ percentile. Approaches to identify influential observations include DFBETAs, which are measures of standardized differences between regression coefficients when a given observation is included or excluded (Choi 2009). Cook’s distance, another method for identifying influential observations, summarizes the influence of each observation on the fitted model parameters after deleting each observation from the estimation and measuring the resulting aggregate changes in estimated costs (Indurkhya et al. 2001).

The goal of this study was to demonstrate, by example, how to identify and handle outliers and how to assess and handle influential observations by measuring their magnitude and impact on colon cancer-related costs (including average episode-based costs and key cost-drivers). This study also compared the effects of statistical costing methods and approaches for overcoming the disproportionate influence of outliers and influential observations.

## Methods

### Study design

We examined data from a retrospective cohort study of veterans aged 66 years or older with colon cancer who were enrolled in both the Department of Veterans Affairs (VA) and Medicare between July 1999 and December 2001. Data included health care use and cost data from the VA; Medicare; eight National Cancer Institute Surveillance, Epidemiology, and End Results (SEER)-affiliated cancer registries; and the VA Central Cancer Registry. A description of a similar cohort is available elsewhere (Tarlov et al. 2012). We excluded patients who had no colon cancer-related costs, were enrolled in a Medicare health maintenance organization, and whose cancer stage at diagnosis was unknown. The final sample comprised 3,842 elderly veterans with stages I-IV colon cancer.

The Edward Hines, Jr. VA Hospital institutional review board (IRB) and the IRBs of the SEER registries approved the study and waived the requirement for informed consent.

### Measures and data sources

We measured colon cancer-related costs in the 12 months following diagnosis and methods are described elsewhere (Hynes et al. 2010). In brief, we classified encounters in Medicare claims and VA records during this period as colon cancer related if they included an International Classification of Diseases, 9^th^ revision, colon cancer diagnosis or colectomy procedure code; Current Procedural Terminology, 4th edition, chemotherapy or colectomy procedure code; Medicare revenue center code; VA outpatient clinic stop code; or Pharmacy Benefits Management (PBM) pharmacy class code for chemotherapy or chemotherapy-related service.

We based costs of services provided through Medicare on payments in institutional inpatient (Medicare Provider Analysis and Review file) and outpatient (Outpatient Standard Analytical File) claims. We also included allowed charge amounts from non-institutional provider claims for care provided under Medicare (Carrier file). We obtained data on costs of care provided through the VA from the Health Economic Resource Center (HERC) Average Cost datasets. HERC estimated average costs for VA inpatient stays using a Medicare cost function estimate developed using patient admission characteristics (Wagner et al. 2003). HERC estimated average costs for VA outpatient visits based on reimbursement rates from Medicare and other health care payers and adjusted these payments to reflect the actual aggregate cost of VA outpatient care (Phibbs et al. 2003). We used VA Fee Basis data (Inpatient, Inpatient Ancillary, and Outpatient files) to identify costs of covered care provided to VA patients outside of VA facilities. Our VA pharmacy costs came from PBM data. The costs we calculated did not include the costs of home health, long-term care (VA only), or hospice care.

We combined the colon-cancer related health care costs for VA and Medicare to determine the costs of a 12-month colon cancer episode of care for each patient in our cohort. We used the Consumer Price Index to adjust these costs to 2004 dollars (Bureau of Labor Statitics and U.S. Department of Labor 2012).

### Approaches to identify outliers and influential observations

We examined four approaches, alone or in combination, for identifying and assessing or adjusting outliers (box-plot analysis and Winsorization) and influential observations (DFBETAs and Cook’s distance) in our full cohort (Tukey 1962; Barnett and Lewis 1994).

The box-plot (interquartile method) is a graphical approach that displays the distribution of data and indicates which observations might be outliers (Pirson et al. 2006). We identified observations from the full cohort as box-plot outliers if ln(cost) > Q3 + 1.5*IQR or ln(cost) < Q1 – 1.5*IQR, where ln refers to the natural logarithm, Q3 is the 75^th^ percentile (upper quartile), Q1 is the 25^th^ percentile (lower quartile), and the interquartile range (IQR) is Q3 – Q1. We used the natural logarithm transformation because the link function we chose for our examination of the GLM models was the logarithmic function.

Winsorization involves replacing (or limiting) extreme values to reduce the effect of outlying values (Thomas and Ward 2006). We Winsorized costs at the 2^nd^ and 98^th^ percentiles by assigning the cost of the 2^nd^ percentile to observations with costs less than that value and by assigning costs of the 98^th^ percentile to costs above that value. In an additional analysis, we Winsorized costs at the 5^th^ and 95^th^ percentiles.

DFBETAs measure, for each regressor in the model, the standardized difference between the regression coefficient when the j^th^ observation is included or excluded. This measurement can be used to determine an observation’s magnitude of influence on each regression parameter estimate. We predicted DFBETA measurements for each regressor in the model. We identified an observation as influential if the absolute value of the predicted DFBETA measurements for stage at diagnosis and colectomy (key cost-driving characteristics) was greater than the size-adjusted cut-off value of 2/√N or 2/√3,842, or approximately 0.03 (Belsley et al. 1980). We also used 0.15 as a cut-off value for identifying an observation as influential because 10–15% change-in-estimate criteria are frequently used to assess confounding in epidemiological studies (Rothman et al. 2008).

Cook’s distance is a technique to measure the aggregate change in the estimated parameter coefficients when each observation is omitted from the estimation and then summarize how each observation influences the fitted model (Indurkhya et al. 2001). We identified observations from the full cohort as influential if their predicted Cook’s distance measurement was greater than the conventional size-adjusted cut-off value of 4/N or 4/3,842 (Fox 1991).

We also considered an observation from the full cohort to be influential and outlying if the predicted DFBETA measurement was greater than 0.15 and the observation was a qualified box-plot outlier.

### Identification and comparison of outlying/influential observations

We calculated the average episode of care cost and distribution for the full cohort. We then identified outlying and/or influential observations using box-plot methods, DFBETAs, and Cook’s distance, and assessed the impact on our calculations of not including these observations. We also adjusted cost values for outlying observations using the Winsorization method. We compared the average costs of each episode of care to those of the cohorts we identified using these methods for handling outliers and influential observations.

### Multivariate analysis

We used multivariate GLM models (gamma family based on modified Park test (Manning and Mullahy 2001) with log link, where ln(E(y|x)) = xβ), to evaluate the association between select key cost-driving characteristics (stage at diagnosis and colectomy) and 12-month colon cancer episode costs of care, while controlling for additional factors. We also performed the GLM modeling using the Poisson and inverse Gaussian families to compare the robustness of our parameter estimates. We calculated estimated expense rate ratios (ERRs) and 95% confidence intervals (CIs). We then compared the key cost-driving variable estimates and the CI widths as a measure of precision from the full cohort to the estimates we obtained after employing the approaches for handing outliers and influential observations described above. Finally, we calculated post-modeling adjusted cost predictions for the key cost-driving variables from the full cohort and we compared these to the cost predictions calculated after we employed the approaches described above.

We used SAS (version 9.3; SAS Institute, Cary, NC) and Stata® MP software (version 12.1, Stata, College Station, TX) for our analyses. Figures were produced using Stata®.

## Results

### Cohort characteristics

**Figure 1:**
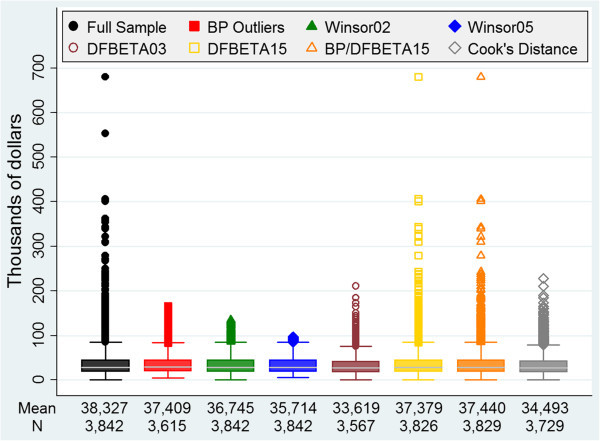
**Sample size, average cost, and cost distribution for each analytic approach.** BP: box-plot.

Among the 3,842 veterans with colon cancer in our cohort who were enrolled in both the VA and Medicare between 1999 and 2001, the average age was 76 years (standard deviation [SD] = 5.7), 96.5% were male, and 15.5% were African American (Table 1). Of these veterans, 26.8% had Stage I, 30.7% had Stage II, 23.2% had Stage III, and 19.3% had Stage IV colon cancer. In addition, 89.4% had undergone cancer-directed colectomy and 33.6% had received chemotherapy within the 12 months following diagnosis. Twenty-three percent had a modified Deyo-Charlson comorbidity score with Romano adaptations of 2 or higher (higher scores indicate a worse baseline health status) (Charlson et al. 1987; Romano et al. 1993; Klabunde et al. 2000; Klabunde et al. 2006). The average cost of colon cancer episodes for the cohort was $38,327 (SD = 37,388), with a range of $43 to $679,472 (Figure 1).

**Table 1:** **Sample characteristics of veterans with colon cancer**

		Full sample
		(N = 3,842)
		%
**Age at diagnosis**	66–75	49.2
	76–85	45.8
	86 and older	5.0
**Gender**	Male	96.5
**Race**	African American	15.5
**Marital status**	Not married	37.7
	Married	59.6
	Unknown	2.8
**Stage at diagnosis**	I	26.8
	II	30.7
	III	23.2
	IV	19.3
**Comorbidity score** ^**a,b**^	0	51.4
	1	25.6
	2–3	18.5
	4 or higher	4.5
**Chemotherapy** ^**c**^	Yes	33.6
**Colectomy** ^**d**^	Yes	89.4
**Hospitals with oncology services per capita**	4^th^ quartile	24.9
**Pre-diagnosis outpatient utilization** ^**e**^	High use	23.5
**U.S. Census division**	New England	3.1
	Middle Atlantic	16.6
	East North Central	9.3
	West North Central	10.4
	South Atlantic	18.9
	East South Central	5.8
	West South Central	13.7
	Mountain	2.8
	Pacific	19.4

^a^Deyo-Charlson Comorbidity Index with Romano adaptations.

^b^Measured from months −6 to 0 in study period.

^c^Measured from months 0 to 12 in study period.

^d^Measured from months −1 to 6 in study period.

^e^Measured from months −6 to −1 in study period.

### Comparisons after the identification of outlying/influential observations

The number of observations we identified as outlying and/or influential varied widely depending on the method we employed.

The box-plot method identified 227 observations as outlying (Table 2). Based on their distribution, 45 observations were upper outlying values and 182 were lower outlying values. Cases identified as outlying using the box-plot method had the lowest average cost ($52,952) of all the methods we used, and the box-plot method identified the second highest number of outlying cases.

**Table 2:** **Summary of costs for observations identified as outliers and influential observations**

	BP outliers	Winsor02	Winsor05	DFBETA03	DFBETA15	BP/DFBETA15	Cook’s distance
	N mean (SD) (range)						
**Overall**							
	227	152	384	275	16	13	113
	52,952	108,152	77,669	99,398	265,093	299,690	164,845
	(113,674)	(128,017)	(94,688)	(94,326)	(117,330)	(98,632)	(104,123)
	(43–679,472)	(43–679,472)	(43–679,472)	(100–679,472)	(50,397–553,115)	(174,413–553,115)	(33,642–679,472)
**Stage at diagnosis**							
I	135	68	167	114	8	6	39
	14,548	49,340	32,973	63,414	230,744	258,473	132,428
	(57,758)	(90,478)	(67,817)	(72,948)	(69,944)	(55,354)	(70,472)
	(77–358,478)	(77–358,478)	(77–358,478)	(100–358,478)	(132,207–358,478)	(213,036–358,478)	(45,404–358,478)
II	40	40	91	62	3	2	41
	112,481	155,546	116,974	136,742	319,201	453,603	172,793
	(166,986)	(150,250)	(110,805)	(119,407)	(253,169)	(140,731)	(127,453)
	(43–679,472)	(43–679,47)	(43–679,472)	(105–679,472)	(50,397–553,115)	(354,092–553,115)	(33,642–679,472)
III	24	22	64	40	2	2	22
	165,703	207,876	134,272	155,595	241,240	241,240	195,733
	(131,600)	(97,587)	(84,274)	(90,093)	(94,508)	(94,508)	(98,996)
	(120–405,892)	(120–405,892)	(120–405,892)	(53,407–405,892)	(174,413–308,068)	(174,413–308,068)	(61,253–405,892)
IV	28	22	62	59	3	3	11
	56,426	104,038	81,940	91,583	318,483	318,483	188,381
	(112,415)	(119,830)	(84,699)	(70,256)	(60,079)	(60,079)	(99,654)
	(71–362,784)	(71–362,784)	(71–362,784)	(362–362,784)	(250,098–362,784)	(250,098–362,784)	(66,227–362,784)
**Colectomy**							
No	73	27	90	110	4	1	14
	5,190	16,293	16,885	34,338	153,281	267,611	100,700
	(31,172)	(59,158)	(45,320)	(44,217)	(89,802)	(−−-)	(61,065)
	(90–267,611)	(90–267,611)	(90–267,611)	(100–267,611)	(50,397–267,611)	(267,611–267,611)	(33,642–267,611)
Yes	154	125	294	165	12	12	99
	75,592	127,993	96,276	142,771	302,364	302,364	173,917
	(130,482)	(130,341)	(98,049)	(93,989)	(102,525)	(102,525)	(105,947)
	(43–679,472)	(43–679,472)	(43–679,472)	(394–679,472)	(174,413–553,115)	(174,413–553,115)	(45,949–679,472)

BP: box-plot.

Winsorization at the 2^nd^ and 98^th^ percentiles replaced 152 observations (76 observations in the lower end and 76 in the upper end; Table 2). By definition, Winsorization at this level replaced 2% of the skewed observations to the right. This method had a middle average cost ($108,152) for the cases identified compared to the other methods. Winsorization at the 5^th^ and 95^th^ percentiles replaced 384 observations (192 observations in the lower end and 192 in the upper end). Winsorization at this level replaced 5% of the skewed observations to the right. The average cost ($77,669) of outlying cases was lower for Winsorization at this level than at the 2^nd^ and 98^th^ percentiles.

The DFBETA method identified 275 observations as influential at the 0.03 cutoff value and 16 at the 0.15 cutoff value (Table 2). The 0.03 threshold, as expected, identified a much larger proportion of influential observations (more than 15 times as many) as the 0.15 threshold. This method identified observations that were influential on both the upper and lower ends, as shown by the lowest cost of all cases identified as influential, $100. The 0.03 threshold resulted in a lower average cost ($99,398) for cases identified as influential compared to the other influential observation methods. The average cost ($265,093) of influential observations identified using the DFBETA method was higher with a 0.15 threshold than a 0.03 threshold, and the minimum cost of influential cases identified using the 0.15 threshold was $50,397.

The Cook’s distance method identified 113 observations as influential using the specified cut-off value (Table 2). Among these influential cases, the average cost ($164,845) was higher than for cases identified with the box-plot and Winsorization methods. The lowest cost of all cases identified as influential by the Cook’s distance method was $33,642.

The method that combined a DFBETA threshold of 0.15 and qualified box-plot outliers identified 13 observations as influential and outlying. Imposing the additional box-plot outlier criterion led to the selection of three fewer cases than the DFBETA method with a 0.15 threshold alone (Table 2). Compared to the other methods, this combined method had the highest average cost ($299,690) for cases identified as influential while identifying the smallest number of influential cases. In addition, the minimum cost, at $174,413, was the highest of all the methods we used.

The average 12-month episode of care costs in the cohorts generated using all of the methods for handling outliers and influential observations were similar (Table 3). The average cost for each colon cancer episode was lowest ($33,619, SD = 22,633, range $43–$210,530) in the cohort generated using the DFBETA method with a threshold of 0.03. The average colon cancer episode cost was highest, at $37,440 (SD = 33,754; range $43–$679,472), in the analysis that combined a DFBETA threshold of 0.15 and qualified box-plot outliers.

**Table 3:** **Summary of costs among cohorts identified using methods for handling outliers/influential observations**

	BP outliers	Winsor02	Winsor05	DFBETA03	DFBETA15	BP/DFBETA15	Cook’s distance
	N mean (SD) (range)						
**Overall**							
	3,615	3,842	3,842	3,567	3,826	3,829	3,729
	37,409	36,745	35,714	33,619	37,379	37,440	34,493
	(25,755)	(27,814)	(23,763)	(22,633)	(33,671)	(33,754)	(24,792)
	(5,310–165,803)	(693–135,659)	(6,255–96,806)	(43–210,530)	(43–679,472)	(43–679,472)	(43–228,199)
**Stage at diagnosis**							
I	893	1,028	1,028	914	1,020	1,022	989
	29,783	26,796	26,437	23,338	26,190	26,428	23,656
	(24,578)	(25,489)	(21,321)	(16,513)	(25,192)	(25,742)	(19,622)
	(5,506–165,803)	(693–135,659)	(6,255–96,806)	(77–116,396)	(77–242,913)	(77–242,913)	(77–160,942)
II	1,141	1,181	1,181	1,119	1,178	1,179	1,140
	36,547	37,074	35,725	33,710	38,406	38,416	34,312
	(25,869)	(27,988)	(23,505)	(22,774)	(38,106)	(38,092)	(24,154)
	(6,315–153,706)	(693–135,659)	(6,255–96,806)	(43–210,530)	(43–679,472)	(43–679,472)	(43–210,530)
III	866	890	890	850	888	888	868
	43,976	45,170	43,750	42,160	46,821	46,821	43,495
	(24,262)	(26,889)	(22,784)	(22,905)	(36,306)	(36,306)	(25,197)
	(7,313–164,668)	(693–135,659)	(6,255–96,806)	(120–184,422)	(120–405,892)	(120–405,892)	(120–228,199)
IV	715	743	743	684	740	740	732
	40,353	39,897	38,909	36,592	39,834	39,834	38,743
	(26,198)	(27,589)	(24,214)	(23,825)	(28,470)	(28,470)	(26,055)
	(5,310–150,985)	(693–135,659)	(6,255–96,806)	(71–150,485)	(71–237,281)	(71–237,281)	(71–150,985)
**Colectomy**							
No	334	407	407	297	403	406	393
	28,911	24,285	24,531	21,071	23,380	24,058	21,947
	(23,990)	(24,383)	(21,417)	(15,166)	(22,543)	(24,147)	(20,392)
	(5,310–162,908)	(693–135,659)	(6,255–96,806)	(90–99,518)	(90–135,659)	(90–162,908)	(90–127,699)
Yes	3,281	3,435	3,435	3,270	3,423	3,423	3,336
	38,274	38,222	37,040	34,758	39,027	39,027	35,971
	(25,775)	(27,829)	(23,681)	(22,855)	(34,377)	(34,377)	(24,849)
	(5,506–165,803)	(693–135,659)	(6,255–96,806)	(43–210,530)	(43–679,472)	(43–679,472)	(43–228,199)

BP: box-plot.

### Multivariate analysis comparisons

**Figure 2:**
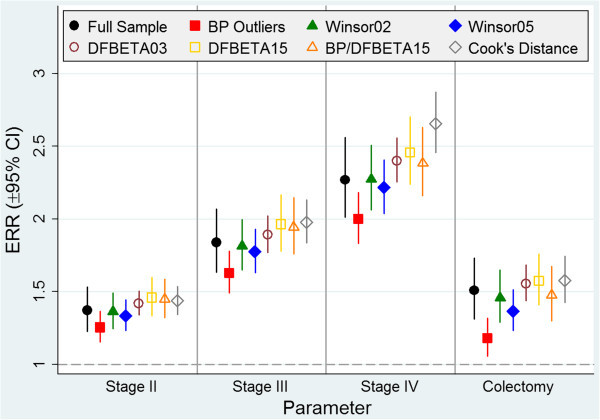
**Estimated expense rate ratios for key cost-drivers.** ERR: expense rate ratio; CI: confidence interval; BP: box-plot.

The GLM regression results using the gamma family (Figure 2) for the full cohort indicate that costs were 51% higher in patients who underwent colectomy (ERR: 1.51, 95% CI: 1.31–1.73) than in those who did not have a colectomy. The colectomy ERRs were similar (range 1.37–1.58) after we employed each of the approaches for handling outliers and influential observations, except for the box-plot method for defining outliers, which resulted in an ERR for colectomy of 1.18. The stage at diagnosis ERRs for the full cohort were of similar magnitude to those obtained with each of the outlier/influential observation methods; the estimates from some of the methods were consistently lower than the estimates from the full cohort and from others were consistently higher. When we examined the CIs for each of the key cost-driving variables, the widths were consistently shortest for the DFBETA method with a threshold of 0.03 and greatest for the method that combined a DFBETA threshold of 0.15 and qualified box-plot outliers.

The parameter estimates generated with GLM modeling using the Poisson family (results not shown) were qualitatively similar to the estimates that resulted from our use of the gamma family GLM (i.e., the stage at diagnosis and colectomy estimates were in the same direction after we used each method for identifying outliers and/or influential observations). The results were also similar quantitatively and of comparable magnitude. All estimates produced from the Poisson modeling were closer than the gamma family estimates to the null hypothesis value, except for the DFBETA method with a threshold of 0.03, which produced estimates for Stage II and Stage IV colon cancer that were further than the gamma family estimates to the null hypothesis value. However, this difference was small.

The results of the GLM modeling using the inverse Gaussian family (results not shown) were also qualitatively similar to the gamma family estimates. The magnitude of the estimates was consistently larger for the inverse Gaussian modeling. All methods for identifying outliers and/or influential observations in the inverse Gaussian modeling were further than the gamma family estimates from the null hypothesis value, except for the full sample, box-plot method, and Winsorization at the 5^th^ and 95^th^ percentiles, whose estimates for stage IV colon cancer were closer than the gamma family estimates to the null hypothesis value. Again, these differences appeared to be negligible.

**Figure 3:**
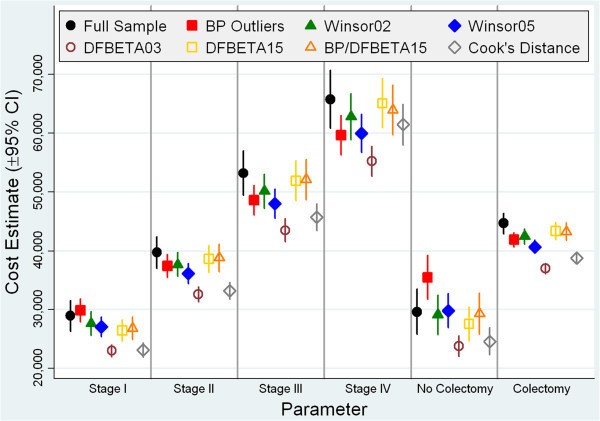
**Estimated Post-Modeling Cost Predictions for Key Cost-Drivers.** CI: confidence interval; BP: box-plot.

Post-modeling predictions revealed that the adjusted costs for patients grouped by stage at diagnosis and colectomy status were consistently lower for each of the methods for identifying outliers and/or influential observations compared to the full sample. Exceptions were the box-plot method, which yielded higher predictions for Stage I colon cancer and patients who did not have a colectomy, and Winsorization at the 5^th^ and 95^th^ percentiles, which yielded higher predictions for patients who did not have a colectomy (Figure 3). Although the ERR estimates were qualitatively similar to one another, the adjusted averages varied depending on the method used. The predicted adjusted average cost that was closest the majority of the time to that of the full sample while selecting the smallest amount of cases came from the method that used a combination of the DFBETA threshold of 0.15 and qualified box-plot outliers.

## Discussion

In this study, we examined four approaches, alone and in combination, for addressing outliers and influential observations in a cohort of 3,842 elderly veterans with colon cancer. The number of observations we identified as outlying and/or influential varied widely depending on the method we employed—from 13 cases when the predicted DFBETA measurement was greater than 0.15 and the observation was a qualified box-plot outlier to 384 cases when we used the Winsorization method at the 5^th^ and 95^th^ percentiles. The average cost of outlying/influential observations ranged from $52,952 with the box-plot method to $299,690 with the combination of a DFBETA threshold of 0.15 and qualified box-plot outliers. But in spite of these differences, the average costs of colon cancer episodes in the cohorts we identified using all of these methods for handling outliers and influential observations were similar.

The variations in the numbers of observations identified as outlying and/or influential by the different methods we employed can be explained by each method’s ability to distinguish between different degrees of skewness to the right. The box-plot method, which identified slightly more than 1% of the skewness to the right, might have overemphasized the lower values of the cost distribution. Similarly, Winsorization might have placed too much emphasis on the lower percentiles of the distribution. The fact that the DFBETA method with the 0.03 threshold resulted in a lower average cost for cases identified as influential compared to the other influential observation methods demonstrates that this method identified more cases on the lower end of the right skewed distribution. However, the fact that the average cost ($265,093) of influential observations was higher and the minimum cost was $50,397 with the DFBETA method and a 0.15 threshold demonstrates that more cases were removed to the right of the population average and that this method selects high leverage values with large residual error. The minimum cost of all cases identified as influential by Cook’s distance, at $33,642, shows that the Cook’s distance method identifies the larger costs of a right skewed cost distribution. Using the method that combined a DFBETA threshold of 0.15 and qualified box-plot outliers resulted in the highest average cost ($299,690) for cases identified as influential, the smallest number of influential cases, and the highest minimum cost for influential cases of all the methods we used. This method targets those observations that are skewed to the right and has a greater than 15% change on the parameter estimate.

All of the methods for handling outliers and influential observations appeared to yield similar results with regard to the average cost estimates. The number of cases that we identified as influential was highest when we used the DFBETA method with a threshold of 0.03, which explains why the calculated mean cost was lowest using the cases in this cohort that we identified. The average colon cancer episode cost was highest, at $37,440 (SD = 33,754; range $43–$679,472), in the analysis that used the combination of a DFBETA threshold of 0.15 and qualified box-plot outliers is due to the fact that the method identified the smallest number of cases. Although these cases were highly influential and outlying, their number was too small to induce a major change in the cost of the average colon cancer episode.

The colectomy ERRs were similar (range 1.37–1.58) after we employed each of the approaches for handling outliers and influential observations, except for the box-plot method for defining outliers, which resulted in an ERR of 1.18. The identification and handling of cases on both the lower and upper ends of the distribution in the box-plot method greatly reduced the margin of difference in cost between the colectomy and no-colectomy cases.

The ERR estimates for stage at diagnosis were consistently lower for the box-plot and Winsorization methods than the ERR estimates for the full cohort. The upper outlying cost values from the cohort identified using the box-plot method had a larger impact on the regression estimates than the lower outlying values because the estimates for each cancer stage were consistently lower than the estimates for each stage in the full cohort even though the box-plot method identified more lower outlying values than higher outlying values. If the patients with the highest costs in our original cohort tended to have Stage III or Stage IV colon cancer, the Winsorization processes were most likely to adjust for these higher costs. As a result, Winsorization consistently yielded stage estimates that were lower than for the full cohort.

Winsorization at the 5^th^ and 95^th^ percentiles resulted in estimates that were lower than Winsorization at the 2^nd^ and 98^th^ percentiles. One likely reason for this was that the method adjusted the higher costs associated with advanced-stage cases to a smaller value (the value of cases at the 95^th^ percentile) than the costs of cases in the 98^th^ percentile, and the costs of Stage I cases, which were lower than the costs of more advanced-stage cases, were adjusted to the cost of cases in the 5^th^ percentile, which was higher than the costs of cases in the 2^nd^ percentile. When we compared the regression estimates of the full sample to the estimates from the box-plot and Winsorization methods, reductions in estimates were generally greater for patients with more advanced-stage cancer because these cases were more likely to be identified as cost outliers.

The regression estimates for stage at diagnosis were consistently higher for the two methods that identified influential costs—DFBETA and Cook’s distance—than for the full cohort. Thus, it is possible that the DFBETA and Cook’s distance methods identified many cases with low or middle costs that were influential in addition to some influential high-cost records, which would increase the regression estimates and gradually increase estimated costs from lower-stage to higher-stage colon cancer.

The method that combined the DFBETA threshold of 0.15 and qualified box-plot outliers produced regression estimates that were very similar to those of the full cohort. A possible explanation might be that, at only 13, the number and value of influential observations we identified using the combined criteria was too small to induce a large change in the model. This method is robust as it uses a combination of outlying and influential criteria and yields results that are consistent with the regression estimates for the full cohort.

We observed that the CI widths were consistently shortest for the DFBETA method with a threshold of 0.03. Even though this method identified the largest number of influential observations, the widths were almost half of the distance compared to the full cohort, indicating that this method produces the greatest improvement in precision. In contrast, the widths were greatest for the method that combined a DFBETA threshold of 0.15 and qualified box-plot outliers. This method identified the smallest number of influential observations, and although it was robust in its targeting of outlying and influential observations, precision was the lowest of all methods.

The similarity of the regression estimates comparing the GLM models using the gamma family to the Poisson and inverse Gaussian families (results not shown) suggested robustness of GLMs in addressing skewness in large datasets. Although we observed this similarity in our data, this might not be the case in all circumstances and careful consideration should be given to successfully specifying the variance function (Manning and Mullahy 2001; Mihaylova et al. 2011).

This study showed that although each of the methods we used identified different numbers of cases as outliers and/or influential observations, these methods produced generally similar overall average costs and average costs by stage at diagnosis and colectomy receipt. Furthermore, the ERRs of the key cost-drivers produced from the GLM modeling were quantitatively and qualitatively similar and of comparable magnitude. However, our post-modeling predictions of average costs for stage at diagnosis and colectomy receipt varied slightly depending on the method we used.

This study compared the effects of using alternative approaches to identifying outlying and influential observations on costs of colon cancer episodes of care. Understanding how estimates could change with each approach is important in determining whether to use a particular method. We used rule-of-thumb cut-off values to identify observations as outlying or influential that are, to some extent, arbitrary, and our findings might have been different if we had used different cut-off values. These remedial measures for handling outliers and influential observations should be employed if the fitted model leads to major changes in the inferences drawn when cases are omitted (Kutner et al. 2004).

## Conclusions

Although we do not recommend any single method for all analyses, we believe that based on the results of this study, the method of choice can be conditionally based on the analytic purpose. If the purpose is to control only for influential observations, then the method of choice is the DFBETA method with a threshold of 0.03 because it produced estimates of similar magnitude to those produced using the full cohort while demonstrating the most improvement in precision as CI widths were consistently shortest. If the purpose is to simultaneously control for outliers and influential observations, then the method of choice is the one that identifies outliers and influential observations using the combination of a DFBETA threshold of 0.15 and qualified box-plot outliers because this method targets those observations that are skewed to the right and has a substantial influence on the parameter estimate. This method produced the closest average colon cancer episode cost and similar regression estimates to those of the full cohort but did so at the expense of precision. The analysis of skewed data should always consider different options for handling outlying and influential cases. Although the conditional methods of choice were applied to cost data in this case, the methods could be appropriate for other data with right skewness as well and the analyst should select an approach for handling outliers and influential observations based on the specific data structure and subject matter knowledge.

## Abbreviations

VA: Department of Veterans Affairs
GLM: Generalized linear model
SEER: Surveillance, Epidemiology, and End Results
IRB: Institutional review board
PBM: Pharmacy Benefits Management
HERC: Health Economic Resource Center
Q1: 25^th^ percentile (lower quartile)
Q3: 75^th^ percentile (upper quartile)
IQR: Interquartile range
ERR: Expense rate ratio
CI: Confidence interval
SD: Standard deviation
BP: Box-plot.
